# Adverse Outcome Pathway Development for Assessment of Lung Carcinogenicity by Nanoparticles

**DOI:** 10.3389/ftox.2021.653386

**Published:** 2021-04-29

**Authors:** Penny Nymark, Hanna L. Karlsson, Sabina Halappanavar, Ulla Vogel

**Affiliations:** ^1^Institute of Environmental Medicine, Karolinska Institute, Stockholm, Sweden; ^2^Environmental Health Science and Research Bureau, Health Canada, Ottawa, ON, Canada; ^3^National Research Centre for the Working Environment, Copenhagen, Denmark; ^4^DTU Health Tech, Technical University of Denmark, Kgs. Lyngby, Denmark

**Keywords:** adverse outcome pathways, nanoparticles, genotoxicity, lung cancer, new approach methodologies

## Abstract

Lung cancer, one of the most common and deadly forms of cancer, is in some cases associated with exposure to certain types of particles. With the rise of nanotechnology, there is concern that some engineered nanoparticles may be among such particles. In the absence of epidemiological evidence, assessment of nanoparticle carcinogenicity is currently performed on a time-consuming case-by-case basis, relying mainly on animal experiments. Non-animal alternatives exist, including a few validated cell-based methods accepted for regulatory risk assessment of nanoparticles. Furthermore, new approach methodologies (NAMs), focused on carcinogenic mechanisms and capable of handling the increasing numbers of nanoparticles, have been developed. However, such alternative methods are mainly applied as weight-of-evidence linked to generally required animal data, since challenges remain regarding interpretation of the results. These challenges may be more easily overcome by the novel Adverse Outcome Pathway (AOP) framework, which provides a basis for validation and uptake of alternative mechanism-focused methods in risk assessment. Here, we propose an AOP for lung cancer induced by nanosized foreign matter, anchored to a selection of 18 standardized methods and NAMs for *in silico*- and *in vitro*-based integrated assessment of lung carcinogenicity. The potential for further refinement of the AOP and its components is discussed in relation to available nanosafety knowledge and data. Overall, this perspective provides a basis for development of AOP-aligned alternative methods-based integrated testing strategies for assessment of nanoparticle-induced lung cancer.

## Introduction

A handful of nanosized particles, including welding fumes, diesel exhaust particles, carbon black, and titanium dioxide (TiO_2_), have been classified as carcinogenic or possibly carcinogenic by the International Agency for Research on Cancer (IARC) (IARC, 2010, 2012a, 2014). Due to lack of epidemiological data for most engineered nanoparticles, insufficient understanding of how their physicochemical properties influence the disease process, and the need for onerous animal-based experimentation, it is not feasible to continue conventional types of risk assessments (Grosse et al., 2014; Catalán et al., 2017; Saber et al., 2019). Thus, the need for reformed safety assessment methods, founded on alternative non-animal approaches, is obvious. Alternative *in silico* and *in vitro* new approach methodologies (NAMs) already exist but are currently applied mainly to identify genotoxic mechanisms and provide plausibility to mutagenic endpoints observed in animals. Promising molecular biology tools such as high-throughput screening of perturbed key molecular players in toxicity pathways and high-content omics profiling are popular but hindered for uptake in safety assessment procedures due to the perceived risk of over-interpretation of the outcome without consideration of the broader biological context and the relevance for human carcinogenicity (IARC, 2014; Catalán et al., 2017).

The current understanding of genotoxic modes of action (MoAs) is fairly far developed and coupled to a battery of more or less standardized assays, including alternative non-animal methods, which have been specifically adapted for testing of nanoparticles (Dusinska et al., 2019). For example, assessment of primary genotoxicity, i.e., the capacity of an agent to produce genetic damage either directly by interacting with DNA or through release of genotoxic reactive oxygen species (ROS) and/or other agents, is supported by a variety of standardized *in vitro* assays, including specifications for applicability to nanoparticles (reviewed in Elespuru et al., 2018; Evans et al., 2019b). Other types of MoAs relevant to nanoparticles, such as secondary genotoxicity, induced by persistent tissue injury and chronic inflammation, has been difficult to address even using animal-based assays and standardized cell-based testing systems do not exist (Catalán et al., 2017). However, recent developments toward advanced physiologically relevant *in vitro* techniques capable of capturing chronic inflammation and secondary genotoxicity by nanoparticles provide solutions through use of co-cultures, conditioned media techniques, and diverse parallel assessments of pro-inflammatory mediators and genotoxicity (Åkerlund et al., 2019; Evans et al., 2019a; Halappanavar et al., 2020b; Kohl et al., 2020). Nevertheless, questions remain as to how to integrate and interpret the resulting data derived from isolated cells in the broader human-relevant context (Dusinska et al., 2017).

Adverse Outcome Pathways (AOPs) offer the much-needed biological context for *in vitro*-derived mechanistic data (Edwards et al., 2016). The AOP framework is conceptually highly similar to the classical MoA concept, which has governed genotoxicity research. However, in contrast to MoAs, which are bound to individual stressors, AOPs are stressor-agnostic and the information contained can be accessed, reused, updated, and applied to a variety of substances (Villeneuve et al., 2014; Sasaki et al., 2020). The AOP framework has emerged as a robust approach for anchoring mechanistic understanding to potential health effects in humans. It is expected to take risk assessment further along toward twenty-first century toxicity testing due to (i) its structured systematic approach including a repository and tools for sharing and collaborating (AOP-Wiki, www.aopwiki.org), (ii) its key focus on mechanistic information, and (iii) its governance by the OECD leading to broad support from large regulatory bodies (Leist et al., 2017). Further details on the framework and its benefits can be reviewed in Leist et al. (2017). The human relevance of the AOP-anchored mechanistic information becomes evident through meticulous integration of diverse types of data (including both legacy and new data) at various levels of biological organization, for the purpose of describing the most central molecular, cellular, organ, and individual level incidents that are connected to the final health effect. AOP development follows a row of central principles and is aligned with well-described guidance documents aiming for particular focus on the *causality* and *essentiality* in the chain of molecular initiating and key events (MIEs and KEs) leading to adverse outcome (AO). In addition, key event relationships (KERs) allow for inclusion of information regarding the required threshold of perturbation for transitioning from one KE to the next (Coady et al., 2019). Thus, the framework also informs and facilitates the development of integrated testing and data interpretation strategies supporting regulatory decision making (Tollefsen et al., 2014; Ede et al., 2020).

Nanotoxicology has recently seen a rise in the development of field-relevant AOPs (Gerloff et al., 2017; Halappanavar et al., 2020b). Worth noting is that although AOPs are stressor-agnostic, they may still be developed through case study approaches, whereby data integration focuses on one or several representative stressors known (or presumed) to be of concern for a specific AO (Gerloff et al., 2017; Halappanavar et al., 2019; Vinken, 2019). Focus within nanotoxicology has been directed toward one of the most relevant target organs for particle exposure, i.e., the lungs, and a set of six AOPs for diverse types of lung injury was recently published (Halappanavar et al., 2020b). In addition, the carcinogenicity of TiO_2_ was recently reviewed leading to a suggested AOP for TiO_2_-induced lung cancer (Braakhuis et al., 2020). The large body of published data and information on nanoparticles provides an extensive basis for further development and refinement of these AOPs (Karlsson et al., 2014; Gerloff et al., 2017; Elespuru et al., 2018; McCarrick et al., 2019).

Here, we describe a putative AOP for lung cancer associated with pulmonary deposition and retention of poorly soluble nanoparticles, covering aspects of both primary and secondary genotoxicity. The case study builds on information from diesel exhaust, carbon black, and TiO_2_ as representative stressors. In addition, a selected set of 18 *in silico* and *in vitro* assays applicable to nanoparticles and available for measurement and assessment of the KEs is aligned with the AOP. While full development of the AOP is beyond the scope of this perspective, we provide insight into the information required and the potential for further refinement of the currently proposed AOP backbone, including examples of available relevant data sets. Worth noting is that this perspective does not cover issues associated with high aspect ratio materials, as the case study stressors do not represent such materials, which have been handled elsewhere (Halappanavar et al., 2020b, 2021).

## Development of a Putative AOP for Lung Cancer Associated With Nanoparticles

**Figure 1:**
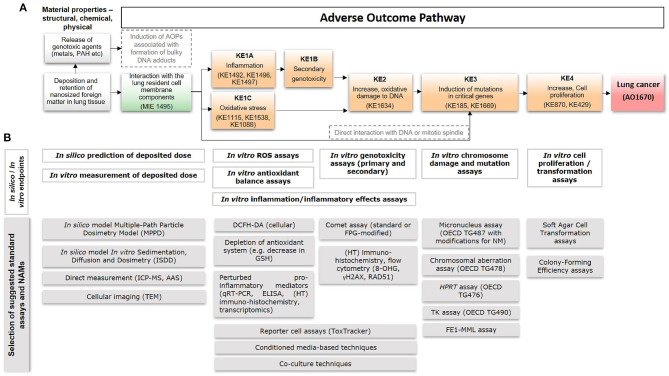
A putative AOP for pulmonary deposition and retention of nanosized foreign matter leading to lung cancer, including anchored *in silico* and *in vitro* methods. **(A)** A putative AOP developed based on information and knowledge about the process-generated and engineered nanoparticles diesel exhaust, carbon black, and TiO_2_. Suggested relevant existing KEs in the AOP-Wiki, that could serve for informing development of the proposed AOP, are mentioned within parentheses. **(B)** The AOP supports integrated application of *in silico*- and *in vitro*-based standard OECD tests with new approach methodologies (NAMs), including models/approaches for prediction of deposited dose, detection of ROS generation, inflammation, DNA damage, mutations, and cell transformation. Examples of specific assays are provided at the bottom. MIE, molecular initiating event; KE, key event; AO, adverse outcome; AOP, adverse outcome pathway; IC-PMS, inductively coupled plasma mass spectrometry; AAS, atomic absorption spectroscopy; TEM, transmission electron microscopy; ROS, reactive oxygen species; DCFH-DA, 2'-7'dichlorofluorescin diacetate; GSH, glutathione; ELISA, enzyme-linked immunosorbent assay; HT, high-throughput; FPG, formamidopyrimidine DNA glycosylase; OECD, Organization for Economic Co-operation and Development; HPRT, hypoxanthine phosphorybosyl transferase; TK, thymidine kinase; FE1-MML, FE1-MutaMouse lung epithelial cells.

The particulate fraction of diesel exhaust is known to be required for carcinogenesis, since filtered exhaust does not cause lung cancer in rodents (Brightwell et al., 1989). Inhaled, nanosized particles deposit primarily in the alveolar region, where clearance is low, and lead to prolonged particle retention enabling particle-bio interaction (Oberdörster et al., 2005; Gaté et al., 2017). Interaction between particles and lung resident cell membrane components (Figure 1A, MIE) leads to inflammation (KE1A) which is proportional to the total deposited surface area (Schmid and Stoeger, 2016; Danielsen et al., 2020; Kokot et al., 2020). The persistence of particles results in long-lasting inflammation (Hougaard et al., 2010; Chézeau et al., 2018). Metabolic activity of pro-inflammatory cells induces formation of ROS, which may also be augmented by the surface reactivity of particles themselves (Jacobsen et al., 2008b; Bendtsen et al., 2020). The sustained inflammatory signaling and concomitant synthesis of reactive radicals, cause a chronic state of oxidant-antioxidant imbalance and loss of protective mechanisms, potentially resulting in secondary genotoxicity (KE1B) (Evans et al., 2019a). Diesel exhaust consists of nanosized particles of inorganic and organic carbon with associated metal oxides and polyaromatic hydrocarbons (PAHs) (Taxell and Santonen, 2016; Bendtsen et al., 2020). Both the carbon core and solvent-extractable fractions containing PAHs are mutagenic *in vivo* and several metal (oxides) have been classified as (possibly) carcinogenic (IARC, 2006, 2012b; Hashimoto et al., 2007). It is possible that such genotoxic agents leach from the pulmonary deposited particles leading to activation of alternative AOPs associated with the formation of bulky DNA adducts and resulting in accumulation of mutations (Li and Nel, 2006) as indicated by the alternative path in Figure 1A (in gray) (Sasaki et al., 2020). In addition, the insoluble carbon core generates particle-induced ROS leading to oxidative stress (KE1C) (Bendtsen et al., 2020; Gren et al., 2020). In a recent study of five diesel exhaust particles designed to differ in chemical composition, DNA strand breaks (KE2) in bronchoalveolar lavage cells were found to correlate with the ROS forming capacity of the particles (Bendtsen et al., 2020). Similarly, carbon black generates surface-dependent ROS, causing oxidative DNA damage (KE2) and mutagenicity (KE3) *in vivo* and *in vitro* (Jacobsen et al., 2008b). Finally, direct interactions between nanosized particles and DNA or the mitotic spindle are also possible, and the AOP features a direct link between the MIE and mutagenicity, i.e., KE3 (Buliaková et al., 2017; Patel et al., 2017).

The genotoxic MoA is less clear for TiO_2_ nanoparticles. However, it is known that the carcinogenic potential of TiO_2_ is highly size-dependent and may include both particle-induced ROS generation and secondary genotoxicity (Liao et al., 2019; Saber et al., 2019). In fact, in 2-year inhalation studies in rats, carbon black, TiO_2_ nanoparticles, and diesel exhaust particles have shown highly similar carcinogenic potency (Saber et al., 2019) and the mutation frequency of carbon black and diesel exhaust particles were also very similar *in vitro* (Jacobsen et al., 2007, 2008a). This may be a coincidence, but it may also indicate that these low solubility particles share common pathways of carcinogenesis. Diesel exhaust, carbon black, and TiO_2_ nanoparticles have all also been shown to induce lung cell proliferation and transformation (KE4) *in vitro* and *in vivo* (Driscoll et al., 1996; Bayram et al., 2006; Medina-Reyes et al., 2015, 2019; Vales et al., 2015).

The MIE, KEs, and AO in the suggested AOP were aligned with relevant existing KEs in the AOP-Wiki, as well as selected evidence for their association with exposure to nanoparticles (Table 1). The table lists the original evidence from the three focus-stressors used to support the development of the AOP (as described above), as well as additional literature associated with metal (oxide) nanoparticles, including nickel (Ni), nickel oxide (NiO), silver, and gold, as well as silicon dioxide (SiO_2_) nanoparticles.

**Table 1:** MIE, KEs, and AO in the suggested putative AOP aligned with existing KEs available in the AOP-Wiki (https://aopwiki.org/) and selected evidence from nanotoxicology literature [with focus on the case study stressors and metal (oxide) nanoparticles].

**KE in newly suggested AOP**	**Existing associated KE in the AOP-Wiki**	**Relevant evidence from studies on nanoparticles**	**References**
MIE	KE1495 interaction with the lung resident cell membrane components	Retained nanoparticles, including TiO_2_, carbonaceous, and a number of metal (oxide) nanoparticles interact with lung resident cell membranes and receptors (e.g., through toll-like receptors)	Chen et al., 2013; Labib et al., 2016; Nikota et al., 2016; Schmid and Stoeger, 2016; Danielsen et al., 2020; Gliga et al., 2020; Kokot et al., 2020; Vasilichin et al., 2020
KE1A	KE1492 tissue resident cell activation	Induction of pulmonary inflammation proportional to the surface area of TiO_2_, polystyrene, carbonaceous nanoparticles, and the insoluble carbon core of diesel exhaust	Hougaard et al., 2010; Saber et al., 2014; Labib et al., 2016; Nikota et al., 2016; Schmid and Stoeger, 2016; Bendtsen et al., 2020; Danielsen et al., 2020; Kokot et al., 2020
	KE1496 increased, secretion of proinflammatory and profibrotic mediators		
	KE1497 increased, recruitment of inflammatory cells		
KE1B	–(Secondary genotoxicity)	Inflammation-driven genotoxicity observed in bronchial epithelial cells for TiO_2_, SPIONs, and NiO nanoparticles	Åkerlund et al., 2019; Evans et al., 2019a
KE1C	KE1115 increased, reactive oxygen species	A number of nanoparticles, including TiO_2_ and SiO_2_, as well as the insoluble carbon core of diesel exhaust generates ROS leading to oxidative stress in lung cells both *in vitro* and *in vivo*	Jacobsen et al., 2008b; Bendtsen et al., 2020; Gren et al., 2020; Karkossa et al., 2021
	KE1538 decreased protection against oxidative stress		
	KE1088 increased, oxidative stress		
KE2	KE1634 increase, oxidative damage to DNA	Diesel exhaust nanoparticles, TiO_2_, and a number of other metal (oxide) nanoparticles, such as silver and gold, cause oxidative damage to DNA in lung cells both *in vitro* and *in vivo*	Karlsson et al., 2014; Golbamaki et al., 2015; Lebedová et al., 2018; Bendtsen et al., 2020; Ling et al., 2020
KE3	KE185 increase, mutations	Diesel exhaust nanoparticles, carbon black, TiO_2_, and a number of other metal (oxide) nanoparticles, such as silver, Ni, and NiO, induce gene mutations, formation of micronuclei, and chromosomal aberrations in lung cells	Jacobsen et al., 2007, 2008a; Golbamaki et al., 2015; Åkerlund et al., 2018; Lebedová et al., 2018; Ling et al., 2020
	KE1669 increased, DNA damage, and mutation		
KE4	KE870 increase, cell proliferation	Diesel exhaust, TiO_2_ and carbon black nanoparticles induce lung cell proliferation, and a number of other metal (oxide) nanoparticles, such as silver, as well as SiO_2_, have been shown to induce lung cell transformation	Driscoll et al., 1996; Bayram et al., 2006; Medina-Reyes et al., 2015, 2019; Vales et al., 2015; Fontana et al., 2017; Gliga et al., 2018
	KE429 cellular proliferation and clonal expansion of mutant cells (pre-neoplastic foci), alteration of cellular growth homeostasis		
AO	AO1670 lung cancer	Diesel exhaust is linked with dose-dependent increase in the risk for lung cancer in humans. Diesel exhaust, TiO_2_, and carbon black are all also coupled to highly similar rates of dose-dependent induction of lung cancer *in vivo*	Mauderly et al., 1994; Heinrich et al., 1995; Vermeulen et al., 2014; Ge et al., 2020

### Factors Contributing to Particle-Induced Lung Carcinogenesis

#### Significance of the Overload Hypothesis

Particle-induced lung cancer in rats has been subject to extensive scientific discussions (Institute IRS, 2000; Saber et al., 2019, 2020) due to the overload hypothesis, which suggests that particle-induced lung cancer observed in rats is an artifact caused by species-specific impaired particle clearance (Warheit et al., 2016). The hypothesis stems from observations that at very high lung burden, particle clearance is completely inhibited in rats, but not in mice (Elder et al., 2005). Furthermore, rats, but not mice, develop lung cancer following particle-exposure (Heinrich et al., 1995). However, particle clearance was demonstrated in inhalation studies showing diesel exhaust-, carbon black- and TiO_2_ nanoparticle-induced lung cancer (Heinrich et al., 1995). The particle clearance half-lives were determined to be 300–600 days (Heinrich et al., 1995), which may be comparable to the half-life of “several hundred days” for humans (Taxell and Santonen, 2016). Dose-response relationship between diesel exhaust exposure and lung cancer occurrence has been determined both in epidemiological studies and in chronic inhalation studies in rats (Heinrich et al., 1995; Ge et al., 2020). Epidemiological evidence indicates that occupational exposure to 1 μg/m^3^ diesel exhaust measured as elemental carbon during a 45-year work life, is associated with 40–170 excess lung cancer cases per 100,000 exposed. Thus, based on epidemiological evidence, lung cancer occurs in humans at exposure levels (1–10 μg/m^3^), where particle overload cannot be a problem. In comparison, 1.3 excess lung cancer cases per 100,000 exposed are expected based on chronic inhalation studies in rats (Vermeulen et al., 2014; Saber et al., 2019; Ge et al., 2020). Hence, when comparing risk estimates based on epidemiological studies with those based on inhalation studies in rats, diesel exhaust appears most potent in the epidemiological studies, indicating that chronic inhalation studies of particles in rats are predictive of human cancer risk, at least with regard to diesel exhaust particles (Saber et al., 2019). The available data on the carcinogenic potency of diesel in both epidemiological studies and animal studies, in addition to knowledge on the potential MoAs in relation to carcinogenicity, makes diesel exhaust a suitable model stressor in the current AOP-development case study.

For TiO_2_, the overload hypothesis in humans remains unsolved due to lack of epidemiological data, as described in the recent review by Braakhuis et al. (2020). Since particle size and hence deposited surface area is a strong determinant of the carcinogenic potency of TiO_2_ particles in animal studies, the lack of information on particle size distribution in available epidemiological studies of TiO_2_ exposure hampers comparison of the carcinogenic potency of these particle in rats and humans (Heinrich et al., 1995).

Taken together, the AOP includes consideration of “Deposition and retention of nanoparticles in the lung” (Figure 1A) and anchors to methods allowing for dose-determination (Figure 1B, see further details on methods below in section Assessment of deposited dose). However, the overload hypothesis is then not specifically considered in the AOP, since literature on diesel exhaust supports the notion that lung cancer in humans is present at lower doses than at which overload would be.

#### Significance of Inflammation and Secondary Genotoxicity

Immune and pro-inflammatory responses are a critical component of host defense (Shacter and Weitzman, 2002). In lungs, the interaction of stressors with resident cells leads to cell injury resulting in release of cellular content such as danger associated molecular patterns or alarmins, including cellular debris, cytokines, and chemokines. Alarmins bind to cell surface receptors and activate inflammatory pathways, such as secretion of a variety of cytokines and chemokines, which in turn signal recruitment of neutrophils, macrophages, and other immune cells to the site of infection or injury (Villeneuve et al., 2018; Halappanavar et al., 2019). The primary purpose of inflammation is to resolve infection and promote healing. However, repeated exposure or tissue persistence of the noxious substance results in unresolved inflammation, leading to tissue injury and chronicity. Chronic inflammation precedes tissue dysfunction and disease progression (Halappanavar et al., 2020a). The immune cells and the pro-inflammatory mediators involved in the inflammatory process are indistinguishable in acute and chronic inflammation, and it is rather the lack of resolution of inflammation, leading to injury, that is potentially causative of cancer. Soluble mediators such as oxidants, arachidonic acid, cytokines, and chemokines released as result of the metabolic activity of immune cells such as neutrophils and macrophages, can lead to oxidative stress, oxidative DNA damage, and cellular proliferation.

Chronic inflammation involving pathogenic infections, chemicals, and non-soluble particles is known to lead to tissue cancer in animal models (reviewed in Shacter and Weitzman, 2002). In addition, particle deposition and persistence, lung inflammation, and increased risk of developing cancer has been observed in workers exposed to coal dust, carbon black, diesel exhaust, and crystalline silica (Kuempel and Ruder, 2009). Poorly soluble low-toxicity particles such as TiO_2_ are suggested to induce cancer via secondary genotoxicity mechanisms involving oxidative stress, chronic inflammation, and cell proliferation (Braakhuis et al., 2020). Furthermore, some engineered nanoparticles induce fibrosis of lungs, which is also linked to initiation of carcinogenesis (Elder et al., 2005; Zhou et al., 2020). Thus, although it has been difficult to find evidence directly supporting the role of inflammation in nanomaterial-induced carcinogenic processes, the existing literature is suggestive of such links. These links have not to date been described in the AOP-Wiki.

### AOP-Anchored Tests for Carcinogenicity Assessment by Nanoparticles

To provide a basis for the development of integrated testing strategies for the assessment of lung carcinogenicity by nanoparticles, we provide an overview of 18 *in silico* and *in vitro* standard methods and NAMs applicable to and in some cases already frequently used for nanoparticles, and anchor them to the putative AOP described above (Figure 1B).

#### Assessment of Deposited Dose

Dose is a key factor for toxicological assessment including for assessment of human relevance, i.e., allowing for comparisons between human and *in vitro* exposure. Exposure via inhalation is typically reported in terms of particle concentration in air (mg/m^3^) whereas *in vitro* studies often report mass of nanoparticles per unit volume (μg/ml). In order to enable comparisons between models, it is helpful to consider cellular target dose as compared to tissue burden. The nominal concentration in the medium of *in vitro* studies can differ substantially from the cell dose since different nanoparticle characteristics affect their ability to reach the cells at the bottom of the culture dish (Teeguarden et al., 2007). For metal (oxide) nanoparticles, the cellular dose can be measured quantitatively using e.g., Inductively Coupled Plasma Mass Spectrometry (ICP-MS) or Atomic Absorption Spectroscopy (AAS), or qualitatively through cellular imaging using Transmission Electron Microscopy (TEM). For ICP-MS and AAS, however, it may be difficult to distinguish between nanoparticles taken up by the cells and those simply attached to the cell membrane. Another limitation is their inability in general to distinguish between the nanoparticle itself and ions released from the nanoparticles. The delivered dose can alternatively be estimated by modeling, e.g., using the *in vitro* Sedimentation, Diffusion and Dosimetry (ISDD) model to estimate the movement of particles to the cells (Hinderliter et al., 2010). Similarly, estimations of tissue deposition of airborne nanoparticles with different characteristics can be performed with the Multiple-Path Particle Deposition model (MPPD), which calculates the deposition and clearance of mono- and polydisperse aerosols containing particles ranging in size from ultrafine/nanosized (100 nm) to coarse (20 μm) in the respiratory tract of humans (Miller et al., 2016). Overall, these methods are useful for assessment and estimation of the deposited dose both *in vitro* and in humans to allow for comparisons and identification of required thresholds of perturbation in results obtained with assays anchored to downstream components of the AOP (Figure 1A).

#### Assessment of Oxidative Stress and Inflammation

Phagocytic cells may internalize deposited particles leading to respiratory burst and release of ROS, leading to oxidative stress and inflammation (KE1C and KE1A, Figure 1A). The fluorometric assay relying on the intracellular oxidation of 2'-7'dichlorofluorescin diacetate (DCFH-DA) is commonly used to detect ROS release in cells *in vitro* (Decan et al., 2016). In addition, lipid peroxidation, protein oxidation, and protein carbonylation can be measured as indicative of oxidative stress using proteomics techniques (Riebeling et al., 2016). Other approaches include measurement of intracellular glutathione levels using the ThiolTracker™ Violet assay (Decan et al., 2016), glutathionylation of proteins, or expression assessment of relevant genes and proteins associated with antioxidant pathways, e.g., using reporter cell lines such as the ToxTracker system (Karlsson et al., 2014; Riebeling et al., 2016).

The selection of pro-inflammatory mediators for investigation differs and is dependent on the expertise of the lab, cell types studied and availability of the specific antibodies. Most routine assays involve measuring the abundance of cytokine mRNA in a given sample using targeted or array-based quantitative (q)RT-PCR. In addition, novel high-throughput and targeted transcriptomic techniques are increasingly becoming available for assessment of transcriptional changes in relevant gene sets and biological pathways (Collins et al., 2017). The protein levels of cytokines and their activation state can also be measured using targeted Western blot assays and ELISA assays. The latter allows quantitative measurement of antigens in biological samples. Both mRNA- and protein-based methods are readily applicable to *in vitro* cell culture models, where cell culture supernatants or whole cell homogenates are useful, and allow for high-throughput, simultaneous assessment of multiple pro-inflammatory mediators in a single setting (Husain et al., 2015). Lastly, immunohistochemistry can be used to detect specific immune cell types producing pro-inflammatory mediators and its downstream effectors in any given tissue (Costa et al., 2018). However, the technique is not quantitative and the sensitivity depends on the specificity of the antibodies employed (Amsen et al., 2009). Some of the most commonly assessed pro-inflammatory mediators include IL-6, IL-8, TNFα, IL-1β, NF-κB, and IFNγ (Nymark et al., 2018a). However, the relative predictive efficiency and sensitivity are not validated and may vary from one test system to another. Moreover, most pro-inflammatory mediators play a pleotropic role and their activities in carcinogenicity still require further research (Gomes et al., 2014).

Recruitment of pro-inflammatory cells is currently not possible to assess *in vitro*. However, with the development of more advanced model systems, such as conditioned media approaches, co-cultures, and ultimately organotypic cultures it may become feasible (Kohl et al., 2020).

#### Assessment of DNA Damage and Mutagenicity, Including Secondary Genotoxicity

*In vitro* assessment of genotoxicity can be divided into two types of assays; (i) assessment of repairable DNA damage or related DNA repair mechanisms, and (ii) assessment of irreparable DNA damage, i.e., inheritable chromosome damage and mutations. Here, we refer to the former as genotoxicity assays, which are anchored with KE1B and KE2 (Figure 1A), and the latter as chromosome damage and mutation assays, anchored with KE3. Detailed definition of genotoxicity and mutagenicity can be reviewed in Catalán et al. (2017).

The comet assay is one of the most commonly used methods for assessing genotoxicity of nanoparticles (Magdolenova et al., 2014; Golbamaki et al., 2015; Elespuru et al., 2018). Overall, the assay is generally applicable to nanoparticles but with some precaution and awareness (Karlsson et al., 2015). For example, photo-catalytically active nanoparticles, e.g., TiO_2_ may cause false positives in the presence of light. In addition, DNA damage formed during the assay performance has also been reported for copper oxide (CuO) nanoparticles, but the extent to which it interferes with the assay remains to be elucidated (Karlsson et al., 2015). The comet assay can also be combined with enzymes, most commonly FPG (formamidopyrimidine DNA glycosylase), allowing for detection of oxidatively damaged DNA (Magdolenova et al., 2014). Recent developments of the comet assay has resulted in higher throughput approach, including automated scoring of mini-gel comets (Jackson et al., 2013; Brunborg et al., 2014; Collins et al., 2017). Other high-throughput assays used for the indication of (still repairable) genotoxicity include e.g., flow cytometry or immunohistochemistry to detect the formation of phosphorylated histone H2AX (γ-H2AX), indicating initiation of DNA repair mechanisms targeting double strand breaks (Nelson et al., 2017; Åkerlund et al., 2018). The ToxTracker reporter assay is also, in addition to oxidative stress mentioned above, capable of assessing genotoxicity, protein folding and p53-related cellular stress based on the transcriptional activation of a set of six key genes (Hendriks et al., 2016). Recent results have demonstrated the applicability of the assay to 33 different nanoparticles, showing a great diversity in activation and magnitude of induction of the various reporters (Cappellini et al., 2020; McCarrick et al., 2020). Thus, these assays have been anchored with KE2 (Figure 1A). Both KE1B and KE2 are also covered by newly developed methods including conditioned media approaches, whereby cells are treated with media from nanomaterial-exposed immune cells, or co-cultures of e.g., macrophages and epithelial cells, as described previously in relation to testing superparamagnetic iron oxide (SPION) and NiO nanoparticles (Åkerlund et al., 2019; Evans et al., 2019b).

With regard to mutagenicity and chromosomal aberration assays, these include another of the most commonly used assay for nanoparticles, i.e., the micronucleus assay [OECD test guideline (TG) 487; Magdolenova et al., 2014; Golbamaki et al., 2015; Elespuru et al., 2018]. Modifications of the assay toward applicability to nanoparticles are included in the standardized guidelines for nanoparticle-application (Elespuru et al., 2018). The assay can be coupled to flow cytometry to increase throughput for scoring of micronuclei (Di Bucchianico et al., 2017; Lebedová et al., 2018). In addition, the Chromosomal Aberration Assay (OECD TG478) is applicable to nanoparticles (Elespuru et al., 2018). Other standardized assays include a variety of mammalian cell mutation assays; the thymidine kinase (TK) assay (OECD TG 490), the *Hprt* (hypoxanthine-guanine phosphoribosyltransferase) assay (OECD TG 476), and the cII loci assay (FE1-MML) (Jacobsen et al., 2007, 2008a; Decan et al., 2016; Åkerlund et al., 2018; Kohl et al., 2020). These have all been anchored with KE3 in the putative AOP (Figure 1A).

#### Assessment of Increased Cell Proliferation and Carcinogenicity

Finally, assessment of cell transformation and increased cell proliferation, as a result of the upstream events, is coupled to cell morphological changes, as shown for e.g., cobalt nanoparticles (Ponti et al., 2009), or the ability of exposed cells to grow in soft agar (Kohl et al., 2020). This is a carcinogenic feature of cells that may be particularly relevant after low dose long-term studies, as suggested for e.g., TiO_2_ nanoparticles, and thus anchored with KE4 (Figure 1A) (Vales et al., 2015). The latest developments of advanced organotypic and 3D models can also be expected to support assessment of the carcinogenic potential of nanoparticles in line with the AO (Kohl et al., 2020).

## Discussion

Assessment of carcinogenicity is coupled to a well-developed set of standardized *in vitro*-based genotoxicity and mutagenicity alternatives that enable assessment of direct-acting carcinogens (Corvi and Madia, 2017). Some of these assays have been or are being evaluated and modified accordingly to be applicable to nanomaterial testing (Karlsson et al., 2014; Nelson et al., 2017). Nevertheless, challenges remain regarding assessment of e.g., secondary genotoxicity MoAs induced by nanoparticles. In addition, there is a need to improve the applicability of new mechanism-focused methods for genotoxicity testing in safety assessment practices. This perspective provides an overview of a putative AOP describing deposition and retention of nanosized foreign matter leading to lung cancer, building on knowledge, and understanding of carcinogenesis induced by diesel exhaust, carbon black, and TiO_2_ nanoparticles. The AOP is aligned with a selected set of *in silico*- and *in vitro*-based standard and new assays which support integrated assessment and interpretation of the putative MIEs and KEs, based on currently available data on nanoparticles (Halappanavar et al., 2020b). Overall, the putative AOP presented here extends on a recently proposed AOP operative for assessment of TiO_2_ carcinogenicity in rats, and opens for the possibility to iteratively build further on the KE descriptions (Braakhuis et al., 2020).

The nanosafety community has over the past decade focused extensively on enabling harmonized data management of the widely diverse data types generated within the field, and has recently led to the establishment of the Nanosafety Data Interface (https://search.data.enanomapper.net/) which provides findable, accessible, interoperable, and reusable (FAIR) data from several European projects (Jeliazkova et al., 2015, in press). Such available data support recently proposed data integration strategies allowing for validation and threshold estimation of the KEs as well as further development of KERs in the suggested AOP (Nymark et al., 2018b; Halappanavar et al., 2021). Eventually, the added value brought by the AOP is particularly coupled to the essentiality and causality between the modules in the chain of events, as well as the AOP-Wiki platform allowing for broad collaborative efforts to gather and store information available for reuse and refinement.

In addition to iterative implementation of knowledge surrounding specific case study stressors, such as here covered by nanosized particles, the development of AOPs also benefits from efforts within other fields, even if challenges remain regarding how to adapt cross-field information to nanoparticles. For example, a recent effort to translate classical genotoxicity MoAs into the AOP framework can be expected to support refinement of the proposed AOP (Sasaki et al., 2020). The work was mainly focused on chemical-induced genotoxic MoAs, however, one of the preliminary AOPs describe the chain of KEs from “increased ROS leading to increases in gene mutations and chromosomal breaks/rearrangement” which is directly in line with KE1C, KE2, and KE3 in the putative AOP suggested here. In addition, the potential for genotoxic agents leaching from nanoparticles and leading to alternative parallel AOPs related to the formation of DNA adducts is supported by these efforts, and it provides a robust basis for further development and branching of AOP networks covering the various carcinogenic MoAs of nanoparticles. Finally, insight from the field of cancer research may provide clues through e.g., the 10 proposed key characteristics of carcinogens and the well-established hallmarks of cancer (Hanahan and Weinberg Robert, 2011; Smith et al., 2016).

Overall, the putative AOP, in its current form, supports systematic and structured integration and evaluation of mechanistic data derived from alternative ways of assessing primary and secondary genotoxicity, supporting safety assessments and prioritization of nanoparticles in need of further testing (Nymark et al., 2020; Sasaki et al., 2020). The evidence gained from systematic application of data derived from these alternative testing approaches may provide insight into further research needs, as well as a robust basis needed for the shift toward safety assessment relying on non-animal methods-driven integrated testing strategies.

## Data Availability Statement

The original contributions presented in the study are included in the article, further inquiries can be directed to the corresponding author.

## Author Contributions

PN conceptualized the idea and wrote the first draft of the manuscript. HK, SH, and UV contributed to development of the putative AOP and to the writing of the manuscript. All authors contributed to the article and approved the submitted version.

## Conflict of Interest

The authors declare that the research was conducted in the absence of any commercial or financial relationships that could be construed as a potential conflict of interest.
